# Do Intervention Programs in Child Care Promote the Quality of Caregiver-Child Interactions? A Meta-Analysis of Randomized Controlled Trials

**DOI:** 10.1007/s11121-015-0602-7

**Published:** 2015-09-28

**Authors:** Claudia D. Werner, Mariëlle Linting, Harriet J. Vermeer, Marinus H. Van IJzendoorn

**Affiliations:** Centre for Child and Family Studies, Leiden University, Leiden, The Netherlands

**Keywords:** Meta-analysis, Child care quality, Intervention, Randomized controlled trials, Child social-emotional development

## Abstract

This meta-analysis reports on the effectiveness of targeted interventions focusing on child care professionals to improve child care quality, caregiver interaction skills, and child social-emotional development. Within randomized controlled trials, interventions are moderately effective in improving overall caregiver-child interactions (*k* = 19, Hedges’ *g* = 0.35) and in improving child care quality on the classroom level (*k* = 11; Hedges’ *g* = 0.39), the caregiver level (*k* = 10; Hedges’ *g* = 0.44), and the child level (*k* = 6; Hedges’ *g* = 0.26). Based on these findings, the implementation of evidence-based targeted interventions on a larger scale than currently exists may lead to better social-emotional development for children under the age of 5 years. There remains, however, an urgent need for more and larger randomized controlled trials with a solid design and high quality measures in order to shed more light on which child care components for which children are most critical in supporting children’s socio-emotional development.

**Electronic supplementary material:**

The online version of this article (doi:10.1007/s11121-015-0602-7) contains supplementary material, which is available to authorized users.

How can the quality of caregiver-child relationships in professional child care be improved? This meta-analysis is the first to focus on randomized controlled trials of targeted interventions in professional child care, focusing on caregiver-child interactions. Professional child care includes home-based care and center-based care (including preschools). Caregivers are formally educated and officially employed to take care of groups of children in the preschool age.

The benefits of enhancing child care quality and preventing child social-emotional problems by implementing effective programs are substantial, given the fact that millions of children under the age of 5 years could be reached. In the USA and most European countries, around 33 % of children under the age of 3 years and around 70 % of children aged 3 to 5 years are enrolled in formal child care (Organization for Economic Co-operation and Development 2013). Therefore, intervention programs for child care that aim at improving caregiver-child interactions require attention. This meta-analysis is highly relevant to the field of preventive intervention science, because knowledge about effectiveness of targeted programs with relatively low costs could provide governments and other funders with vital background information for their investments.

Empirical studies have shown that more positive caregiver-child interactions in professional child care are related to fewer behavior problems and higher cognitive-academic achievement in primary school (Belsky et al. 2007; Peisner-Feinberg et al. 2001). In many western countries, there is still much debate on how to deliver high quality care (Chambers et al. 2010) and how to find a balance between the costs and benefits of intervention programs, especially since more and more children are attending child care (Barnett 2011; Chambers et al. 2010; Nelson et al. 2003; Weikart 1998). Different routes have been taken to improve child care quality: (1) Early Childhood Education (ECE) programs and (2) targeted intervention programs.

## ECE Programs

Bronfenbrenner’s ecological model (1979) formed the basis of many broad-focus child care programs, also referred to as ECE programs. The core assumption of the model is that children are affected by experiences in multiple social environments simultaneously, which all contribute to child development. The focus is broad in the sense that the programs focus on improvements by targeting various social environments at once, involving child care providers, parents, and teachers, to reach optimal outcomes (Ramey and Landesman Ramey 1998). The programs are generally long-term (several years rather than weeks) and most of them aim at disadvantaged children from low SES families (Hungerford and Cox 2006). Well-known ECE programs in the USA are the High/Scope Perry Preschool program (Belfield et al. 2006), the Carolina Abecedarian (Campbell et al. 2012), the Chicago Longitudinal School Readiness program (Jones et al. 2013), and Head Start (Shager et al. 2013). Most of these programs have focused on child cognitive development (Blok et al. 2005; Burger 2010; Nelson et al. 2003), and mixed results regarding child outcomes have been reported (Barnett 2011; Clarke and Campbell 1998; Nelson et al. 2003). It is difficult to disentangle effective aspects when many program components and “players” (e.g., parents, teachers, and trainers) are involved. It should be noted that within some ECE programs great efforts have been made to systematically examine program effects using control groups and randomized assignment of participants (Campbell et al. 2012; Belfield et al. 2006). Impressive long-term advantages of ECE programs up to the age of 40 years have been shown regarding psychological well-being, employment, and non-criminal behavior (Campbell et al. 2012; Belfield et al. 2006).

## Targeted Interventions

The goal of this meta-analysis is to evaluate the intervention programs in child care settings that target only one “ecological environment” at a time. Such targeted interventions may be part of ECE-programs, but can also stand alone. Examples include all training methods for caregivers and children in child care settings that are aimed at achieving specific goals within that setting. Targeted interventions are generally short-term, i.e., they are completed within a time span of several weeks or months. Moreover, these programs are generally easier to implement and allow researchers to study the effectiveness of particular aspects of the program, because of the narrow focus and the single environmental setting.

It has been suggested that targeted interventions may be as effective on the childs’ cognitive and social-emotional domains as ECE programs (Barnett 2011; Burger 2010; Nelson et al. 2003). However, studying the effectiveness of targeted interventions may still be a complex task given the variation in how programs are implemented and how program fidelity is ensured. Due to a lack of well-designed experimental evaluations in this area (Barnett 2011; Burger 2010), it is yet unclear whether targeted interventions actually succeed in improving child-care quality. When investigating this effectiveness, several levels of quality indicators should be considered: the classroom level, the child level, and the caregiver level (Please note that in this paper groups in home-based child care are also referred to as classrooms.). At the classroom level, quality of care may be indicated by the amount of space in the room, play materials, hygiene practices, social atmosphere, and/or general supervision of children (Harms et al. 1998; Riksen-Walraven 2004). At the child level, child care quality can be indicated by social-emotional wellbeing and peer interaction (Riksen-Walraven 2004). Finally, at the caregiver level, caregiver interaction skills are important quality indicators (Harms et al. 1998; Riksen-Walraven 2004).

## Importance of Caregiver Interaction Skills

Whereas child-focused trainings aimed at improving child cognitive school readiness are widespread, the caregiver-child relationship and child social-emotional development as targets of intervention in child care have been less thoroughly investigated (Blok et al. 2005; Chambers et al. 2010). Yet, for very young children, caregiver-child interactions are highly important, because caregivers can provide them with feelings of security and may stimulate their development (Burchinal et al. 2002; Vermeer and Bakermans-Kranenburg 2008). Caregivers thus play a crucial role in children’s social-emotional development. Addressing this role, Riksen-Walraven (2004) defined six important caregiver interaction skills for professional caregivers: sensitive responsiveness, respecting children’s autonomy, structuring and limit setting, enhancing verbal communication, stimulating peer interaction, and developmental stimulation. These skills can be assessed from caregiver practices, attitudes, beliefs, or knowledge about caregiving. The question arises how these caregiver interaction skills can be improved. With respect to intervention programs, one might ask whether training-on-the-job, that is, additional caregiver training, may improve caregiver interaction skills even further, in order to enhance child social-emotional outcomes (Burchinal et al. 2002; Howes et al. 1992). However, researchers have struggled to reach consistent conclusions about effective elements of caregiver training, because of a wide variety in focus, scale, and design of the programs (Fukkink and Lont 2007). With regard to their design, outcomes of interventions on caregiver-child interaction skills and child social-emotional competence have been reported far less often in randomized trials than cognitive school readiness programs (Blok et al. 2005; Burger 2010; Chambers et al. 2010).

Over the past decade, several meta-analyses on early childhood interventions in child care have been published (Blok et al. 2005; Fukkink and Lont 2007; Nelson et al. 2003). However, it remains difficult to distill clear conclusions on the effectiveness of targeted interventions for child care quality and child outcomes. For instance, Blok et al. (2005) and Nelson et al. (2003) included both targeted interventions and broad-focus ECE programs. Moreover, they focused not specifically on caregiver interaction skills, but on child emotional well-being, parent–child relationships (Nelson et al. 2003), or education (Blok et al. 2005). Second, outcomes were not reported on the classroom level, but on the child level (Blok et al. 2005; Nelson et al. 2003) or the caregiver level (Fukkink and Lont 2007). Therefore, no conclusions can be drawn on the effectiveness of programs on the classroom level, an indication for general quality of care. Finally, the three meta-analyses were restricted by including quasi-experimental studies, potentially confounding internal validity issues with conclusions about the effectiveness of the programs.

## Research Objectives

We focus on the effectiveness of targeted interventions focusing on child care professionals in improving child care quality at the classroom level, caregiver interaction skills, and child social-emotional development. Our study is different from previous meta-analytic reports in the field in at least two ways: (1) it only includes randomized controlled trials of targeted interventions and (2) beyond reporting overall effectiveness of the programs, results are reported separately for the three levels that represent child care quality: the classroom level, the caregiver level, and the child level. Because the targeted interventions focus on behavioral changes in the caregiver, we expect effect sizes at the caregiver level to be higher than effect sizes at the classroom and child level. We conduct a meta-analysis to answer the main research question. In addition, we investigate which aspects of the care settings and the intervention programs may moderate the effectiveness of interventions in professional child care.

## Moderators

In our moderator analysis, we make a distinction between characteristics of the intervention and characteristics of the child care setting. Regarding intervention characteristics, we focus on possible differential effects dependent on the focus of the intervention, the duration and intensity of the programs, the type of sessions that are provided to caregivers (group sessions, individual sessions, or both), and the use of video, because meta-analyses have shown that these may be important moderators (see, e.g., Bakermans-Kranenburg et al. 2003; Blok et al. 2005; Fukkink and Lont 2007; Nelson et al. 2003)*.* Because effect sizes may be different depending on the types of treatment for the control group, we will also distinguish between placebo versus no placebo control groups. We expect higher effect sizes in studies using placebo control groups (see Blok et al. 2005).

Child care moderators are investigated in a more explorative way. First, the type of child care setting may matter. Center child care and preschool are both arrangements with relatively large groups and multiple caregivers, whereas home-based care is more similar to the family setting, with only one caregiver present in a home-like environment and generally no more than eight children in one group (Rusby et al. 2004). We will further distinguish intervention programs that were conducted in the context of subsidized Head Start settings, because Head Start centers share the same standard program and serve a specific group of children (from low income families).

Furthermore, some studies may not only involve caregiver training but also child curricula implemented by the caregiver. Child curricula generally consist of weekly activities around a certain theme that are described in a detailed activity manual for caregivers. The caregiver leads the activities, for instance, by inviting the children to discuss theme-related topics during circle time, role play, or storybook reading. The child curriculum may be the basis of some intervention programs, whereas the caregiver training part may be small. In other programs, there may be no specific child curriculum, leading activities to be less structured, less frequent, or not directed at the group of children as a whole, but the caregiver training part may be more extensive. We will examine in an explorative way whether the use of these child curricula affects the outcomes of the interventions.

## Method

### Literature Search

**Fig. 1:**
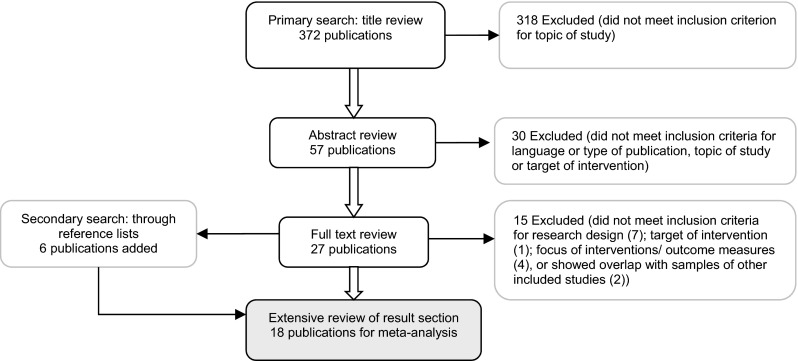
Flow chart of study selection process

To identify relevant studies, the following electronic databases were systematically searched for articles with any starting date and published until 2013: Web of Science, SCIRUS, PsychInfo, WorldCat, ERIC, Google Scholar and Dissertation.org. The following keywords were entered: *child care*, *daycare*, *preschool*, *center-based care*, *home-based care*, *family-based care*, in combination with one or more of the following keywords: *intervention*, *staff training*, *teacher training*, *caregiver training*, *and child development.* Subsequently, the reference lists of collected studies were searched for relevant studies. A flow chart of the literature search is shown in Fig. 1. To be included in the meta-analysis, articles had to meet the following inclusion criteria: (1) the study design was a randomized controlled trial, (2) the language of publication was English, (3) the study was published (or available online) as an article in a research journal, or a doctoral thesis, (4) the topic of study was an intervention or training, targeting professional caregivers, or teachers for typically developing children aged 0 to 5 years in professional child care. The intervention may encompass the implementation of a standard curriculum for the children, if it was in combination with or through caregiver/teacher training. In addition, (5) the article should report on at least one of the following outcomes considering child care quality: general quality of the child care setting measured at the classroom level (e.g., classroom atmosphere, support and instructions from teacher to the group, or ratings of conflicts between children, as long as these constructs are measured at the group level); caregiver interaction skills as indicated by caregiver practices, attitudes, beliefs, or knowledge about caregiving (measured for individual caregivers); or quality at the child level (measured for individual children), as indicated by child social-emotional development, or child communication skills that are used and needed for social interaction, such as verbal interaction and responsiveness to questions. Finally, (6) information provided in the results section should allow for calculation of effect sizes for outcome measures.

Considering the first criterion, a control group and randomized assignment to groups were required. Studies reporting placebo interventions for the control group were allowed, but only if the contents of these placebo interventions were unrelated to the programs in the intervention groups. Considering criterion 4, studies were excluded when they focused on caregivers for preschool children beyond the targeted age range, e.g., children in kindergarten, or if the intervention program targeted children directly, i.e., not through training of the caregiver. Community-based programs including parental involvement were also excluded. Regarding the outcome measures, studies were excluded if the intervention was exclusively aimed at aspects other than listed in criterion 5. For instance, at the classroom level programs targeting physical environment for health and hygiene practices were excluded. At the child level indicators of cognitive school readiness, such as alphabet knowledge, phonological awareness and mathematical skills were excluded (e.g., excluding Downer et al. 2011). Following these criteria, 18 articles were included (see Fig. 1). The studies were reviewed and coded by two independent coders. Inter coder reliability was satisfactory with 0.70 or higher (range 0.71 to 1.00) for both categorical variables (kappa) and numerical variables (intra class correlation) (Fukkink and Lont 2007; Nelson et al. 2003). When information was reported unclearly, consensus was reached between the two coders through discussion.

### Coding System

The studies were coded on study and intervention characteristics. We coded year of publication, publication outlet, country of the study, and research design, by distinguishing pretest- posttest design and posttest-only design. An overview of the most important characteristics of the studies is presented in Table 1. The most relevant aspects of the intervention programs are presented in Table 2. For the type of care, three categories were used: preschool, center-based care, and home-based care. To distinguish clearly between different levels of randomization and outcomes, we refer to centers as well as preschools as “centers”; to caregivers (in home-based care or center-based care) as well as teachers in preschools as “caregivers”; and to groups (for groups in home-based care and classrooms in center-based care and preschools) as “classrooms”. The level of randomization was coded on the highest level: district level, center level, classroom level, caregiver level, or child level. Sample sizes (differentiated by intervention and control group) were coded on all levels for pretest and posttest. Regarding the characteristics of the intervention programs, we coded the name of the program, number and duration (in hours) of intervention sessions, and whether these were group sessions or individual sessions. We calculated the total number of hours of the intervention (individual sessions and group sessions apart and their sum) and the duration of the total intervention period (in months). If the authors only reported that the intervention lasted for “a school year”, we coded the intervention period as 8 months. The time between pretest and posttest was coded (in months). In case authors reported that “pretests were carried out at the beginning of the school year and posttest at the end of the school year”, we coded the time between pretest and posttest as 9 months, calculating 1 month extra on top of the time coded for the duration of the intervention. In addition, we coded the use of an additional child curriculum (yes or no), the use of video (video-feedback, video-modeling, or no video), and the “treatment” for the control group (placebo intervention, waitlist, or care-as-usual).

**Table 1:** Sample characteristics

Study	Country	*SES of the children*	*Type of care*	N of centers		N of classrooms		N of caregivers		N of children		Level of randomization	Outcome level(s)
				I	C	I	C	I	C	I	C		
Baker-Henningham et al. (2009)	Jamaica	Low SES	Preschool	3	2	15	12	15	12	22 per classroom		Center	Class, caregiver
Barnett et al. (2008)	USA	Low SES	Preschool	1 in total		7	11	7	11	81	117	Child	Class, child
Cain et al. (2007)	USA	NR	Center	27 in total		NR	NR	16	19	NR	NR	Caregiver	Caregiver
Domitrovich et al. (2007)	USA	Low SES: Head Start	Preschool	NR	NR	10	10	NR	NR	100	100	Center	Child
Domitrovich et al. (2009)	USA	Low SES: Head Start	Preschool	NR	NR	22	22	42	42	NR	NR	Center	Class, caregiver
Driscoll and Pianta (2010)	USA	Low SES: Head Start	Preschool	29 in total		NR	NR	NR	NR	38	40	Center	Child
Fukkink and Tavecchio (2010)	The Netherlands	NR	Center	NR	NR	NR	NR	52	43	NR	NR	Center	Caregiver
Girard and Girolametto (2011)^a^/Girolametto et al. (2004)	Canada	NR	Center	4	3	NR	NR	8	9	32	36	Center	Caregiver/child^a^
Girolametto et al. (2003)	Canada	NR	Center	2	2	8	8	8	8	32	32	Center	Caregiver
Groeneveld et al. (2011)	The Netherlands	NR	Home	–	–	24	24	24	24	7 per caregiver		Caregiver	Class, caregiver
Izard et al. (2004)	USA	Low SES: Head Start	Preschool	NR	NR	7	9	NR	NR	58	58	Caregiver	Child
Neuman and Cunningham (2009)^b^	USA	Low SES	Center (PD)	53	35	53	35	53	35	NR	NR	Caregiver	Class
Neuman and Cunningham (2009)^b^	USA	Low SES	Center (PDC)	53	35	53	35	53	35	NR	NR	Caregiver	Class
Neuman and Cunningham (2009)^b^	USA	Low SES	Home (PD)	32	22	32	22	23	22	NR	NR	Caregiver	Class
Neuman and Cunningham (2009)^b^	USA	Low SES	Home (PDC)	34	22	34	22	34	22	NR	NR	Caregiver	Class
Raver et al. (2008)^a^/Zhai et al. 2011 ^a^	USA	Low SES: Head Start	Preschool	9	9	18	18	48	42	455 in total		Center	Class/caregiver^a^
Rusby et al. (2004)	USA	NR	Home	–	–	18	20	18	20	NR	NR	Caregiver	Class
Rusby et al. (2008)	USA	NR	Home	–	–	18	20	33	30	8.7 per caregiver		Caregiver	Class
Snyder et al. (2011)	USA	Low SES: Head Start	Preschool	3	2	9	5	18	10	76	33	Center	Caregiver, child

*N* number at posttest, *I* Intervention group, *C* control group, *NR* Not reported, *PD* professional development, *PDC* professional development plus coaching

^a^Two publications report on one study but on different outcome levels

^b^Publication covers two settings and two intervention programs

**Table 2:** Characteristics of intervention programs and study designs

Study	Name of the program	*Focus*	*Program duration (months)*	*Pretest to posttest (months)*	*Group training total hours)*	*Individual coaching (total hours)*	*Use of child curriculum*	*Use of video*	*Treatment control group*
Baker-Henningham et al. (2009)	IY Teacher Training	SR	7.00	9.00	56.00	14.00	Yes	Modeling	Placebo
Barnett et al. (2008)	Tools of the mind	SR	7.00	9.00	41.00	20.00	Yes	No	Placebo
Cain et al. (2007)^a^	Focus-Follow-Talk	SR	3.00	–	4.00	2.25	No	No	Placebo
Domitrovich et al. (2007)	PATHS	VC/PI	9.00	9.00	24.00	NR	Yes	No	Waitlist
Domitrovich et al. (2009)^b^	PATHS-REDI	VC/PI	12.00	13.00	34.00	160.00	Yes	Modeling	Care-as-usual
Driscoll and Pianta (2010)	Banking Time	SR	1.50	NR	NR	NR	No	No	Waitlist
Fukkink and Tavecchio (2010)	Video Interaction Guidance	SR	NR	NR	0.00	4.00	No	Feedback	Care-as-usual
Girard and Girolametto (2011)^c^/Girolametto et al. (2004)^c^	Learning Language & Loving it	VC/PI	1.50	2.00	7.00	1.50	No	Modeling + feedback	Placebo
Girolametto et al. (2003)	Learning Language & Loving it	VC/PI	3.00	4.00	20.00	3.00	No	Modeling + feedback	Waitlist
Groeneveld et al. (2011)	VIPP-SD	SR	5.00	6.00	0.00	9.00	No	Feedback	Placebo
Izard et al. (2004)	Emotions Course	VC/PI	5.00	7.00	2.00	4.00	Yes	No	Care-as-usual
Neuman and Cunningham (2009)^d^- center based	Professional Development	VC/PI	3.50	8.00	45.00	0.00	No	Modeling	Waitlist
Neuman and Cunningham (2009)^d^ -home based	Professional Development	VC/PI	3.50	8.00	45.00	0.00	No	Modeling	Waitlist
Neuman and Cunningham (2009)^d^—center based	Professional Development + Coaching	VC/PI	8.00	8.00	45.00	48.00	No	Modeling	Waitlist
Neuman and Cunningham (2009)^d^—home based	Professional Development + Coaching	VC/PI	8.00	8.00	45.00	48.00	No	Modeling	Waitlist
Raver et al. (2008) ^c^/Zhai et al. (2011)^c^	IY Teacher Training	SR	7.00	8.00	38.00	45.00	No	Modeling	Placebo
Rusby et al. (2004)	Carescapes—1 module	SR	0.03	1.00	3.00	0.00	No	Modeling	Waitlist
Rusby et al. (2008)	Carescapes—3 modules	SR	1.50	2.50	9.00	0.00	No	Modeling	Waitlist
Snyder et al. (2011)	IY Teacher Training	SR	3.00	8.00	15.00	2.25	No	No	Care-as-usual
Total	0.35	0.07	0.21	0.48	<0.001				

Moderators in italic

^a^Posttest-only design

^b^Only posttest results reported

^c^Two publications report on one study but on different outcome levels

^d^Publication covers two settings and two intervention programs

*NR* Not reported, *SR* Sensitive Responsiveness, *VC* Verbal communication, *PI* Peer interaction

### Sample Description

The included articles (*n* = 18) were published between 2003 and 2012. None of the relevant articles published before 2003 reported on a randomized controlled trial. There was overlap between studies in two cases. Girolametto et al. (2004) and Girard and Girolametto (2011) reported on the same sample, but on different outcome levels. In a different study, the same holds for Raver et al. (2008) and Zhai et al. (2011). Neuman and Cunningham (2009) reported in one publication on two intervention programs in two settings, providing four study samples. Finally, 19 study samples were distinguished from 16 different intervention studies. We clustered the studies according to the focus of the intervention, using the description of caregiver skills provided by Riksen-Walraven (2004). Nine studies targeted mainly caregiver sensitive responsiveness, and most of these also included aspects of respecting children’s autonomy, structuring, and limit setting. Seven studies were mainly focused on enhancing verbal communication and peer interaction. Developmental stimulation defined as stimulation of physical or cognitive development was not the main focus of any of the interventions described here, because for the current meta-analysis we selected studies that aimed at improving caregiver-child interactions and children’s socio-emotional development. In Appendix 1, we briefly describe the theoretical background and goals of the intervention programs.

### Meta-analytic Procedures

We conducted a meta-analysis using the Comprehensive Meta-Analysis software (Borenstein et al. 2000, 2009). Four analyses were conducted: one on the database as a whole and three sub-analyses on subsets of the database, focusing on the three separate levels of outcome measures (classroom level, caregiver level, and child level). Within these four sets of studies, we calculated Hedges’ *g*, a variant of Cohen’s *d* that is more appropriate for small samples.

The first set of studies considered the overall effect size of the randomized controlled trials on social interaction of caregivers and children. To ascertain similarity of outcome measures, we excluded variables not reflecting social behavior or interaction, and very specific caregiver aspects, such as stress and job satisfaction. One study (Zhai et al. 2011) was not included because the only reported outcome considered caregiver reported job stress.

The second, third, and fourth subsets of studies included studies on the classroom level, caregiver level, and child level, respectively. However, it was not possible to use “outcome level” as a moderator in the overall meta-analysis, because the majority of studies reported on more than one outcome level. Therefore, separate datasets were created on the three different levels and three additional meta-analyses were conducted. Within the three separate datasets, outcomes were meta-analytically combined using CMA, which leads to a conservative estimate of the overall effect size (Borenstein et al. 2009).

Random-effects models were applied, accounting for sampling error between as well as within studies. A random-effects model allows for the possibility that there are random differences between studies that are associated with variations in procedures, measures, settings, that go beyond subject-level sampling error and thus point to different study populations. Homogeneity of the sets of effect sizes was tested with Q-statistics. It should be noted that in this meta-analysis and additional analyses the number of studies was relatively small (ranging from *k* = 6 to *k* = 19), so that interpretation of the Q-value as an indication of the homogeneity of outcomes should be done with caution, another reason to use the random model.

### Trim-and-fill Method

We used the trim-and-fill method to calculate the effect of potential data censoring or publication bias on the outcome of the meta-analysis. Using this method, a funnel plot is constructed of each study’s effect size against the sample size or the standard error (usually plotted as 1/*SE* or precision). If no publication bias was present, this plot would show the shape of a funnel, because studies with smaller sample sizes and larger standard errors are expected to have increasingly large variation in estimates of their effect sizes as random variation becomes increasingly influential, whereas studies with larger sample sizes have smaller variation in effect sizes (Duval and Tweedie 2000; Sutton et al. 2000). However, since smaller non-significant studies are less likely to be published, studies in the bottom left hand corner of the plot are often absent. With the “trim and fill” procedure, the *k* right most studies considered to be symmetrically unmatched are trimmed and their missing counterparts are imputed or “filled” as mirror images of the trimmed outcomes. This leads to an adjusted estimate of the combined effect size taking into account potential publication bias.

### Reported Statistics

Regarding reported statistics, the following decisions were made. We based the analyses as much as possible on raw data (pre- and post-means, standard deviations, and sample size). However, none of the studies, except the study by Groeneveld et al. (2011), reported on correlations between pre- and posttest outcomes. Therefore, we used a default estimate of 0.50 for the pre-post correlations, which is in accordance with the empirical values reported by Groeneveld et al. (2011) (i.e., 0.43. and 0.56). An estimate of 0.50 was also applied in the meta-analysis by Fukkink and Lont (2007). When means and standard deviations were reported in combination with an *F*-value for the interaction, but no correlation between pre- and posttest, we entered the data with *F*-values to avoid uncertainty about the pre-post correlation. When reported sample sizes differed for pretest and posttest, posttest sample sizes were used. When only regression coefficients were reported, we selected the unstandardized *b*-values, corrected for pretest score and as few as possible covariates. When only significance levels were reported instead of exact *p* values, we used the significance levels as a conservative estimate of the *p* values. In one study, two different treatment effects were investigated in two different contexts: home-based care and center-based care (Neuman and Cunningham 2009). We analyzed this sample as four separate studies, equally dividing the control group across experimental groups within each context to prevent individuals from being included more than once in the meta-analysis. Regarding the robustness of the effect sizes, we applied Rosenthal’s criterion, implying that if the fail safe number is larger than 5 *k* +10 (with *k* the number of studies in the meta-analysis), it can be concluded that the effect size might be rather robust.

### Moderator Analysis

In the overall meta-analysis, we investigated the role of potential moderators. It should be noted that moderator analyses will only be performed if a subset consists of at least four studies (*k* ≥ 4), as has become established convention (Bakermans-Kranenburg et al. 2003). Considering program characteristics, we compared interventions that used video (aggregating in one variable the use of individual video-feedback and/or video modeling, *k* = 12) with those without video (*k* = 7) and programs including a child curriculum (*k* = 5) versus those without (*k* = 14). We compared program duration, distinguishing between shorter than 6 months (*k* = 11) and longer than 6 months (*k* = 7). For one study, duration was not reported. The intensity, that is the total amount of hours dedicated to training (group training and individual training combined), was categorized as less than 10 h (*k* = 7) and 10 h or more (*k* = 11). The number of individual training hours was not reported in two studies, so that total training hours could not be calculated. Furthermore, we distinguished programs with individual training sessions (irrespective of the number of hours) (*k* = 15) from those without (*k* = 4). We used Riksen-Walraven’s model (2004) to compare programs by their focus of intervention: caregiver sensitive responsiveness (*k* = 9) versus caregiver verbal communication and stimulating peer interaction (*k* = 10). Also, we regarded treatment for the control group as a moderator, distinguishing two categories: placebo (*k* = 6) versus no placebo (*k* = 13). The no-placebo category included care-as-usual and waitlist, similar to Blok et al. (2005). With respect to the type of care, we compared center-based (including preschools, *k* = 14) versus home-based settings (*k* = 5) and programs within Head Start settings (*k* = 6) versus those without Head Start (*k* = 13).

## Results

### Overall Effect

The combined effect of the 19 randomized controlled studies with combined outcome measures on all levels was Hedges’ *g* = 0.35 (SE = 0.07), CI = 0.21–0.48, *p* < 0.001) and there was no indication for heterogeneity (Q = 22.50, *p* = 0.21). The fail-safe number was 171, indicating that 171 studies with null results would be needed to reduce the overall significant effect to non-significance. After applying the trim and fill method, the adjusted effect size was Hedges’ *g* = 0.25 (CI = 0.10–0.40, Q = 40.96), including six trimmed studies. The necessity to trim studies pointed into the direction of publication bias against small studies with small effect sizes (Borenstein et al. 2009). The effect sizes of the studies are presented in Table 3.

**Table 3:**
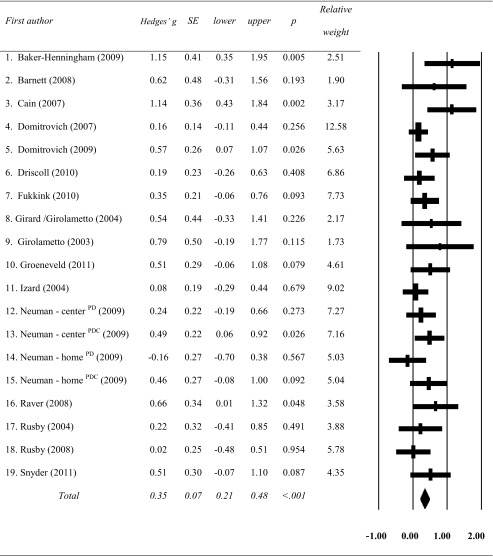
Overall outcomes on social-interaction measures from RCTs in child care: effect sizes (Hedges’ *g*, 95 % confidence interval) and significance

*PD* Professional development group, *PDC* Professional development plus coaching group

### Effects on the Classroom Level, Caregiver Level, and Child Level

The combined effect of 11 randomized controlled studies with outcome measures on the classroom level was Hedges’ *g* = 0.39 (*SE* = 0.10), CI = 0.19–0.59, *p* < 0.001) and there was no statistical indication for heterogeneity (Q = 13.56, *p* = 0.19). The fail-safe number on the classroom level was 52. After applying the trim and fill method, the adjusted effect size was Hedges’ *g* = 0.33 (CI = 0.11–0.54, Q = 20.09), including two trimmed studies. The effect sizes on the classroom level are presented in Appendix 2 Table 4.

For the caregiver level, the combined effect of ten randomized controlled studies was Hedges’ *g* = 0.44 (*SE* = 0.12), CI = 0.21–0.68, *p* < 0.001) and there was no statistical indication for heterogeneity (Q = 13.43, *p* = 0.14). The fail-safe number on the caregiver level was 48. After applying the trim and fill method, the adjusted effect size was Hedges’ *g* = 0.30 (CI = 0.04- 0.55, Q = 24.95), including three trimmed studies. The effect sizes on the caregiver level are presented in Appendix 2 Table 5.

For the child level, the combined effect of six randomized controlled studies was Hedges’ *g* = 0.26 (*SE* = 0.08), CI = 0.11–0.40, *p* = 0.001) and there was no statistical indication for heterogeneity (Q = 0.70, *p* = 0.98). The fail-safe number on the child level was 11. After applying the trim and fill method, the adjusted effect size was Hedges’ *g* = 0.24 (CI = 0.10–0.38, Q = 1.25), including one trimmed study. Effect sizes on the child level are shown in Appendix 2 Table 6.

### Moderator Analysis

We investigated the role of potential moderators. No moderator effects were found for the type of care (Q = 1.52, *p* = 0.22), Head Start versus no Head Start (Q = 0.67, *p* = 0.41), use of video (Q = 0.06, *p* = 0.80), use of a child curriculum (Q = 0.07, *p* = 0.79), program duration (Q = 1.60, *P* = 0.21), and focus of intervention (Q = 1.16, *p* = 0.28). No moderator effects were found for overall program intensity: programs with less than 10 h in total were not significantly different in their effectiveness than programs with 10 or more hours of training (Q = 0.750, *p* = 0.39).

The presence of an individual training component was a significant moderator (Q = 4.198, *p* = 0.040). Programs with individual training sessions for caregivers led to higher effect sizes (Hedges’ *g* = 0.41 (*SE* = 0.07), CI = 0.27–0.55, *p* < 0.001) than programs without individual training (Hedges’ *g* = 0.09 (*SE* = 0.14), CI = −0.18–0.36, *p* = 0.52). Treatment for the control group was also a significant moderator (Q = 9.431, *p* = 0.002), showing that programs with a placebo training for the control group (Hedges’ *g* = 0.75 (*SE* = 0.15), CI = 0.46–1.05, *p* < 0.001) were more effective than programs without a placebo treatment (Hedges’ *g* = 0.25 (*SE* = 0.06), CI = 0.12–0.37, *p* < 0.001).

### Power Analysis

We performed a priori power analyses with G*power 3.1 (Faul et al. 2007), calculating the sample size needed to detect the aggregated effect size (i.e., the assumed population effect size) with a power of 0.80 and a two-sided significance level of 0.05. For the overall meta-analysis on outcomes related to social behavior and interaction (aggregated Hedge’s *g =* 0.35), a minimum sample size of *N =* 260 would be needed. Note that for the trim-and-fill adjusted effect size of *g* = 0.25, we would even need more than 500 subjects. Post hoc power analysis showed that the power of the included studies to detect the aggregated effect size ranged from 0.10 for the study with the smallest sample size (Girolametto et al. 2003; *N* = 16, combined reported effect size *g* = 0.79) to 0.69 for the study with the largest sample size (Domitrovich et al. 2007; *N* = 200, combined reported effect size *g* = 0.16). We also performed power analyses considering the meta-analytic outcomes on the classroom, caregiver, and child level separately. Results showed that we would need 210 classrooms, 166 caregivers, and 468 children to detect the aggregated effect sizes of 0.39, 0.44, and 0.26, respectively. On all three levels, post hoc power to detect the aggregated effect size was far below the required 0.80, the highest value being 0.56 (Fukkink and Tavecchio 2010; *N* = 95; caregiver outcomes with combined reported effect size *g* = 0.33).

## Discussion and Conclusions

In this meta-analysis, we showed that targeted interventions focused on caregiver-child interactions are moderately effective in improving child care quality on three levels: classroom quality, caregiver interaction skills, and, to a lesser extent, child behavior. Thus, the implementation of targeted interventions may lead to higher child care quality, and eventually, better social-emotional development for children under the age of 5 years. According to Cohen’s criteria, the reported effects are small to medium. For our overall meta-analysis, which should be considered the most important one, we found a rather robust effect size. The significant effect sizes on the three separate levels are based on fewer studies, resulting in larger confidence intervals. Specifically, when we also take into account possible unpublished studies (with trim-and-fill), the effect sizes become rather small and relatively unstable. Even so, meta-analysis has the advantage compared to a narrative review that it is replicable and takes trends in primary studies into account in computing combined effect sizes. We consider it informative that caregiver training seems to be indeed most effective for quality at the caregiver level, and less for quality at the classroom level and child level.

Although it is remarkable that only in the last 10 years randomized controlled trials on targeted interventions have been published, it is promising to see a shift towards more solid research designs in the field of child care and early childhood education. Still, there is much room for improvement. For instance, the studies included in our meta-analysis were systematically under-powered as a result of the small number of subjects. Moreover, in many studies, we were confronted with lack of information needed to perform a meta-analysis, for instance, sample size, randomization procedures, raw data (means and standard deviations), and pretest-posttest correlations). In addition, we were confronted with missing information on intervention characteristics such as duration and spacing of training sessions (e.g., Driscoll and Pianta 2010; Domitrovich et al. 2007; Fukkink and Tavecchio 2010). Furthermore, although the studies were rather homogeneous in design, they were at the same time heterogeneous with regard to other aspects such as sample size, SES of the sample, focus of the intervention programs, duration and frequency of training sessions, and outcome measures. The relatively small set of studies (*k* = 19) restricted our exploration of identifying effective components within and between studies.

Our moderator analyses showed that results do not differ depending on child care characteristics (home-based versus center-based; Head Start versus no Head Start). Considering program characteristics, we found no effects of use of video, use of a child curriculum, program duration, focus of intervention, and program intensity. A remarkable moderator was the presence of placebo training for the control group. Programs without a placebo intervention were less effective, which is in contrast with Blok et al. (2005). It is possible that the studies with a placebo intervention for the control group do not report more effective programs, but merely represent methodologically higher quality studies with better outcome assessments. Our conclusion that overall program duration and intensity did not moderate program effectiveness should be considered with caution, because the small number of studies forced us to dichotomize these moderators in our analyses. Our findings are in line with those of Blok and colleagues (2005), but in contrast with those of Nelson et al. (2003) who concluded that more lengthy and more intense programs in preschool are more effective. It should be noted that studies in the meta-analysis by Nelson et al. (2003) directly targeted children, not caregivers.

Unfortunately, we were unable to test the “less is more” hypothesis which states that short-term intervention programs with relatively few sessions are more effective than long-term programs with many sessions (Bakermans-Kranenburg et al. 2003). In our meta-analysis, it was not possible to distinguish exact numbers of training sessions, because of missing data and variation across studies in type (group and/or individual sessions), contents, and duration of sessions. Instead, we distinguished studies with and without an individual training component. We cautiously conclude that there seems to be added value of individual coaching on top of group training sessions. However, the small number of studies did not allow us to further analyze whether intensity of the individual training component also matters. It would not be surprising that individual attention for the caregivers leads to improvement of their skills. Still, it is important to have better understanding of the minimal dose that is needed for individual training, so that costs can be reduced.

Some limitations of the current meta-analysis should be mentioned. First, the number of pertinent studies is rather small which restricts moderator analyses and prevented us from conducting multivariate meta-regression. Second, we found evidence for publication bias that might have led to inflated estimates of effect sizes. With the trim-and-fill method, we have tried to create a more valid estimate. Third, the studies included in the meta-analysis were underpowered which might reflect on the overall meta-analytic outcome.

Despite these limitations, we suggest taking the current findings as a tentative description of the current state-of-the-art that shows the promising nature of targeted interventions in child care. In fact, in the meta-analytic literature, one might differentiate between two types of meta-analyses, discovery-oriented versus confirmation-oriented meta-analytic studies. In the first type of discovery-oriented meta-analysis, fruitful hypotheses for further inquiry are generated (e.g., see Van IJzendoorn 1995; 14 studies). In the confirmation-oriented meta-analyses, the empirical state of the art with respect to a theory or set of hypotheses is presented, with a temporary closure of the discussion (Bar-Haim et al. 2007). Here, we present meta-analytically derived hypotheses that may lead future preventive intervention studies in this area.

### Implications for practice and future research

An important conclusion for the field and policy makers is that focused training programs to improve caregiver interaction skills are moderately effective. Although children were not directly targeted in the intervention programs described here, they do seem to benefit from these types of trainings. The effect on the child level regarding child social-emotional behavior was small, yet significant. Our findings implicate that programs in child care with a relative short term and therefore possibly relatively low-costs can be effective in preventing child problem behavior. In future studies, cost-effectiveness of targeted interventions and ECE programs should be investigated so that they can be compared. Funders and authorities may want to reconsider their current prevention programs or caregiver trainings to improve child care quality. However, there is a need for more, and especially larger, randomized controlled trials. Well-designed intervention studies may teach us what critical components for which children and families are most critical in terms of socio-emotional development. Only when numerous studies are conducted using solid designs with sufficient power and high quality measures can we start to advise policy makers which evidence based programs to implement to increase child care quality. Evidence on effectiveness of relatively low-cost interventions is essential for governments and other funders and may guide decisions on future investments. Effective targeted interventions could then start to play a key role in improving the wellbeing of many young children in professional child care.

## Electronic supplementary material

ESM 1 (DOCX 25 kb)

## Figures and Tables

**Table 4:**
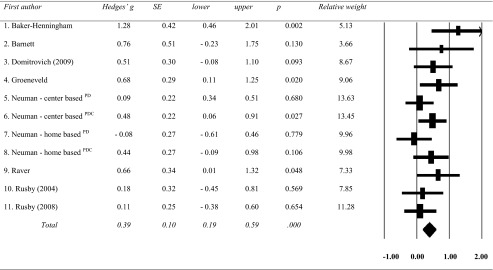
Classroom level outcomes from RCTs in child care: effect sizes (Hedges’ *g*, 95 % confidence interval) and significance

*PD* Professional development group, *PDC* Professional development plus coaching group

**Table 5:**
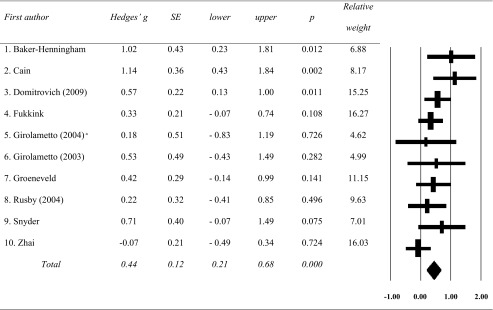
Caregiver level outcomes from RCTs in child care: effect sizes (Hedges’ *g*, 95 % confidence interval) and significance

^a^Outcomes on child level of this study reported by Girard et al. (2011)

**Table 6:**
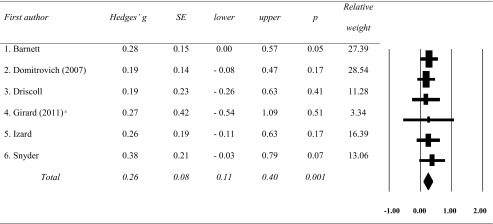
Child level outcomes from RCTs in child care: Effect sizes (Hedges’ *g*, 95 % confidence interval) and significance

^a^Outcomes on caregiver and classroom levels of this study reported by Girolametto et al. (2004)
