# Supplementary material for: Do Intervention Programs in Child Care Promote the Quality of Caregiver-Child Interactions? A Meta-Analysis of Randomized Controlled Trials
Source: Prev Sci. 2015 Sep 28;17:259–73. doi: 10.1007/s11121-015-0602-7 (PMC4718933; doi:10.1007/s11121-015-0602-7)
Supplement: Supplementary file 1 — (DOCX 25 kb) [file 11121_2015_602_MOESM1_ESM.docx]

**Appendix 1**

Original name, authors, background and aims of the programs evaluated in studies included in current meta-analysis.

| **Name of original program** | **Program evaluated by** | **Goal and underlying theory** |
| --- | --- | --- |
|  |  | ***Focus on sensitive responsiveness*** |
| *Banking Time*  (Pianta & Hamre, 2001) | Driscoll et al. (2010) | Based on the assumption that caregiver-child relationships serve as resources for children to enhance development and emotion-regulation skills. A key aspect is non-directive interaction between caregiver and child. In a one-on-one setting caregivers practice observing the child’s actions and describing them aloud, and labeling the child’s feelings and emotions. Relational themes are brought up by the caregiver, to build a supportive relationship. |
| *Carescapes – Setting up to Support Social Development* (Rusby et al., 2004; Rusby et al., 2008); *Proactive approaches for managing children’s behavior, Understanding and dealing with problem behavior* (Rusby et al., 2008) | Rusby et al. (2004)  Rusby et al (2008) | The underlying theory is that caregiver-child relationships and child social competence can be promoted by improving the physical environment. Topics of discussion are how a safe and orderly environment, the arrangement of the physical space, selection of materials and toys, and setting schedules and routines may elicit children’s social skills. In two additional modules structuring and limit setting were added to caregiver target behavior. |
| *Focus-Follow-Talk*  (Cain et al., 2007) | Cain et al. (2007) | To improve joint attention in caregiver-child interactions, which is seen as an important precursor for social-emotional skills and language development. Caregivers are coached to focus on the object of the child’s interest, follow the child’s lead by engaging in non-interfering ways and talk about the object in ways that promote language development. |
| *Incredible Years Teacher Training Program (IYTTP)*  (Webster-Stratton, 2004) | Baker-Henningham et al. (2009)  Raver et al. (2008)  Snyder et al. (2011)  Zhai et al. (2011) | Aim is to prevent and reduce child problem behaviors. Caregivers are trained to use labeled praise to promote positive behavior, and to use clear rules and routines to improve the caregiver-child relationship and child social-emotional skills. Stress-reduction workshops for caregivers and mental health coaching for children are available in adapted versions. The child curriculum consists of 14 weekly lessons of 30 minutes: Circle time discussions, songs, role play and group activities, with the goal to capture children’s anger management, their recognition of emotions, and the use of school rules. |
| *Video Interaction Guidance* | Fukkink & Tavecchio (2010) | Program rooted in a somewhat general communication theory implying that positive caregiver-child relationships enhance child social-emotional development. During video guidance sessions, there is a focus on caregiver’s interactive behaviors such as turning towards the child, making eye contact, recognizing the child initiatives, following the child, and acknowledge actions of the child and oneself. |
| *Video-feedback Intervention to Promote Positive Parenting and Sensitive Discipline (VIPP-SD)*  (Juffer, Bakermans-Kranenburg, & Van IJzendoorn, 2008) | Groeneveld et al. (2011) | Originally developed for families and adapted to a version suited for child care. The program is rooted in Bowlby’s (1969) attachment theory and Patterson’s (1982) coercion theory. The caregivers are trained to recognize children’s signals of exploration and contact seeking behaviors, to take the child’s perspective, and to use empathy, induction and praise in order to set limits in a sensitive manner. |
|  |  | ***Focus on verbal communication and peer interaction*** |
| *Early Childhood Experiences in Language Arts*  (Machado, 2003) | Neuman & Cunningham (2009) | The program stresses the importance of child care providers’ knowledge of child language and literacy development, as well as general interaction skills. Caregivers are advised how to ask children open-ended questions, elicit discussions, and support interaction. |
| *Emotions Course*  (Izard, 2001) | Izard et al. (2004) | The program is rooted in Emotion Theory. The author reasons that improvements in emotion knowledge and regulation will lead to pro-social behavior, because children learn how to communicate and empathize from their emotions. The program stresses self-regulation and aims to improve child social-emotional development by enhancing communication skills. The child curriculum consists of 22 lessons, including activities with puppets, storybooks, posters and games to foster emotion recognition, expression and utilization. |
| *Learning Language and Loving It – The Hanen Program for Early Childhood Educators*  (Weitzman, 1994) | Girard et al. (2011)  Girolametto et al. (2004)  Girolametto et al. (2003) | Caregivers are trained to follow the child’s lead, encourage turn-taking, and use responsive labels, thereby expanding and extending the children’s use of language. |
| *Promote Alternative Thinking Strategies (PATHS)*  (Domitrovich, Greenberg, Kusché, & Cortes, 2005) | Domitrovich et al. (2007)  Domitrovich et al. (2009) | The rationale is that by improving awareness, expression of emotions, and self-regulating skills in young children, problem behaviors can be prevented. The program was extended by adding *Research-based Developmentally Informed* (REDI) components, including interactive reading activities, sound games and alphabet activities to improve caregiver strategic use of language. Stimulation of verbal communication by the caregiver is emphasized. Additional goals are to improve child vocabulary, narrative, and syntax. The child PATHS curriculum consists of 30 weekly lessons, including circle time activities and games, art projects and storybook reading. |
| *Tools of the mind*  (Bodrova & Leong, 1996) | Barnett et al. (2008) | Vygotsky-based program that aims to improve child language skills, social-emotional development and self-regulation. Key elements are the caregivers’ scaffolding behavior and facilitation of learning opportunities. A child curriculum of 40 weekly activities is the core of the program, including circle time activities, dramatic play, and elements to enhance self-regulation, memory, cognition, and attention. |

References

Bowlby, J. (1969). *Attachment and loss* (Vol. 1). *Attachment*. New York, NY: Penguin Books.

Bodrova, E., & Leong, D. J. (1996). *Tools of the mind: The Vygotskian approach to early childhood education.* Upper Saddle River, NJ: Prentice-Hall.

Domitrovich, C. E., Greenberg, M. T., Kusché, C., & Cortes, R. (2005). *The preschool PATHS curriculum*. South Deerfield, MA: Channing Bete.

Izard, C. E. (2001). *The Emotions Course. Helping children understand and manage their feelings: An emotion-centered primary prevention program for Head Start. Teachers Manual.* Unpublished manuscript, Newark, DE: University of Delaware.

Juffer, F., Bakermans-Kranenburg, M. J., & Van IJzendoorn, M. H. (2008). *Promoting Positive Parenting: An attachment-based intervention*. New York, NY: Lawrence Erlbaum Associates.

Machado, J. (2003). *Early childhood experiences in the language arts* (7^th^ ed.). New York, NY: Delmar.

Patterson, G. R (1982). Coercive family processes. Eugene, OR: Castilia.

Pianta R. C., & Hamre, B. (2001). *Students, teachers, and relationship support (STARS).* Lutz, FL: Psychological Assessment Resources.

Webster-Stratton, C. (2004). *The Incredible Years Teacher Training Series*. Seattle, WA: Incredible Years.

Weitzman, E. (1994). The Hanen program for early childhood educators: In-service training for child care providers on how to facilitate children’s social, language and literacy development. *Infant-Toddler Intervention: The Transdisciplinary Journal, 4*, 173-202.
